# Building the evidence base on the HIV programme in India: an integrated approach to document programmatic learnings

**DOI:** 10.1186/s12961-018-0291-3

**Published:** 2018-03-12

**Authors:** Deepika Ganju, Bidhubhusan Mahapatra, Rajatashuvra Adhikary, Sangram Kishor Patel, Niranjan Saggurti, Gina Dallabetta

**Affiliations:** 1 HIV and AIDS Program, Population Council, 142 Golf Links, New Delhi, 110003 India; 2 UNAIDS, Formerly with Population Council, New Delhi, India; 3 Bill & Melinda Gates Foundation, Seattle, USA

**Keywords:** HIV programme, Evidence, India, Documentation, Mentorship, Peer-reviewed publications, Scientific writing

## Abstract

**Background:**

The Knowledge Network project was launched in 2010 to build evidence on the HIV epidemic by using the data generated by HIV programme implementing organisations in India. This paper describes the implementation of the programme and the strategies adopted to enhance the capacity of individuals to document and publish HIV prevention programme learnings. Further, it discusses the outcomes of the initiative.

**Methods:**

A multipronged approach was adopted, where a group of experts were brought together to collaborate with programme implementing organisations, review available data, develop research questions and guide peer-reviewed publications. Further, scientific writing courses were conducted to support individuals from HIV programme implementing organisations as well as educational and government organisations (mentees) to build the documentation capacity of individuals leading programme implementation and current and future researchers. The impact and quality of evidence generated was measured by examining the number of papers published, the number of citations, and the number of papers with at least 10 citations. Additionally, course participants’ responses to open-ended questions in the anonymous course evaluation questionnaires are presented as verbatim quotes.

**Results:**

Overall, 99 papers on HIV programmatic learnings from India were finalised under the programme, of which 95 have been published. In all, 67 papers were co-authored by mentees. Most papers were published in high-impact factor (1 or more) journals and 72% were cited at least once in the literature. The main themes documented include key populations’ HIV risk, HIV risk of general population groups, HIV/STI service delivery models and community mobilisation interventions.

**Conclusion:**

The study demonstrates that an integrated approach, involving partnership, capacity-building and mentorship, can maximise the use of available data and build the evidence base on HIV programmatic learnings. The capacity-building model adopted in the programme can be used to build scientific writing and documentation capacity in other public health programmes that are implemented at scale.

**Electronic supplementary material:**

The online version of this article (10.1186/s12961-018-0291-3) contains supplementary material, which is available to authorised users.

## Background

Scientific evidence is necessary to identify and prioritise health needs and to inform policy, programmes and services for better health outcomes [1]. In India, there has been a focus on generating evidence to guide the national HIV programme since 1992, when the Indian government launched the first phase of the National AIDS Control Programme (NACP I) under the National AIDS Control Organisation (NACO) [2]. Following the scale-up of prevention efforts during NACP III (2007–2012), a vast amount of surveillance, programme and evaluation data was collected by multiple stakeholders, including government, national and international agencies, universities and programme implementing organisations, spanning wide geographies, a large population base and diverse intervention environments on a range of critical issues.

While NACO’s priority during NACP III (2007–2012) was to maximise the use of available data for evidence-based planning [3], studies suggest that the wealth of data on the HIV epidemic in India available at the time was not being rapidly translated into evidence in the form of peer-reviewed publications [4–8]. An assessment of HIV/AIDS-related publications worldwide indicated that in 2002–2003, just 1.4% of the publications were from India, although India had approximately 10% of the world’s estimated HIV-positive population in 2002 [5]. In health research more generally, India’s relative contribution was also low; over a 10-year period (1992–2001) India contributed just 12 out of 1000 published articles worldwide [7].

Assessments at the time have also highlighted the limited evidence base on key issues for HIV programming in India [5, 6, 8]. Published research focused mainly on basic and clinical sciences, while publications in critical areas, such as the validation of HIV estimation approaches, epidemiology of HIV in high-risk groups, impact evaluations of HIV prevention programmes for high-risk groups and the cost-effectiveness of programmes, were poorly represented. Moreover, evaluation studies were based on relatively small sample sizes [5, 6, 8].

In the context of the availability of a vast amount of data on the HIV epidemic in India, the paucity of evidence-based publications and limited documentation capacity in the country [6, 9], and to align with the national priority of building evidence to inform HIV programme implementation, in 2010, the Population Council launched the Knowledge Network project. This is an innovative programme to maximise the use of data and build sound evidence on the HIV prevention programme in India, which could guide future interventions in India and other settings with similar epidemics. The programme specifically aimed to strengthen national capacity to document HIV prevention programmatic learnings from India and to disseminate evidence to wide audiences through the publication of evidence-based papers in peer-reviewed journals. This paper describes the implementation of the documentation programme and the strategies adopted to enhance the capacity of individuals to document and publish HIV prevention programme learnings, and discusses the outcomes of the initiative.

## Methods

### Context

In the context of the growing HIV epidemic in India, several organisations have been implementing large-scale HIV prevention interventions since 2003 across the six high-HIV prevalence states of India – Maharashtra, Tamil Nadu, Andhra Pradesh, Karnataka, Manipur and Nagaland. In the process, a vast amount of theme-specific and programme-related data was collected through a robust monitoring system [10]. However, this data was limited to programme use, primarily to guide programme decisions and activities related to resource allocation, implementation scale-up, course corrections and shifts in implementation, programme redesign, impact evaluation and advocacy, and were not being published as evidence [11]. Against this background, the Knowledge Network project adopted an integrated approach to document the different HIV programme strategies being implemented, synthesise learnings from multiple datasets on the HIV programme, and support the documentation of HIV prevention programme lessons from across the country.

### The Knowledge Network project

The Knowledge Network project was implemented from 2010 to 2016. Given that a vast amount of survey data was available, the project sought to ensure that the data were maximally utilised for scientific output beyond the primary purpose of programme monitoring. It provided the producers of data (programme managers) an opportunity to use the programme data and document their experiences. Simultaneously, by providing datasets to young investigators and making data available for triangulation, it sought to reduce the ‘hold on data’, and increase data sharing and hence the use of data for scientific research.

The project adopted a multipronged approach to build the evidence on the HIV epidemic in the country. For one, it brought together a core group of experts from six research and academic institutes to review the available data and develop research questions in the context of national and global research priorities, and publish papers on identified themes. At the same time, it trained individuals from HIV programme implementing organisations and young researchers in scientific writing and mentored them to document and publish HIV programme lessons from India in peer-reviewed journals within a predetermined timeframe (the capacity-building and mentorship process is discussed in the next section). Project funding also supported Council research teams to analyse the data and publish papers on identified gaps and priority documentation areas. Additionally, in partnership with NACO, the project built the documentation capacity of over 40 National Data Analysis Plan (NDAP) analysts through scientific writing workshops, and supported analysts to finalise and publish programme data as peer-reviewed papers. Finally, 13 workshops were conducted on the principles of scientific writing, data analysis, interpretation and presentation, and documentation in collaboration with educational and government organisations (Tata Institute of Social Sciences, NACO and the Family Welfare Training and Research Centre, Government of India, among others) to build the documentation capacity of over 400 current and future researchers working in the field of HIV and public health, including Masters’ and Doctoral students, faculty from national universities and medical institutes, and Maharashtra State AIDS Control Society (MSACS) programme staff (project activities and outputs are presented in Table 1).

**Table 1:** Capacity-building and mentorship programme for documentation and publication of evidence-based papers: Activities and outputs

Training in scientific writing and mentorship (2010–2013)	
Number of capacity-building and mentorship courses conducted	Three courses comprising an initial training workshop in scientific writing and topic conceptualisation, followed by workshops and small group meetings to revise and finalise draft papers^a^
Number of mentees trained over three courses	70
Number of dropouts (did not complete the workshop)	None
Training in scientific writing and mentorship (2014–2015)	
Number of scientific writing workshops conducted for NDAP analysts (in partnership with NACO)	Three workshops, with follow-up support for paper writing
Number of NDAP analysts trained	40
Workshop sessions	
Principles of effective writing, selecting a title, writing the abstract, writing the introduction, reviewing the literature, writing the methods section, data analysis (qualitative and quantitative), presenting data, writing the results and writing the discussion, principles of authorship, publication ethics and addressing reviewers’ comments; training in reference manager software	
Outputs (2010–2016)	
Number of abstracts received for paper writing	110
Number of programme-supported publications^b^	99^c^

^a^See Fig. 1 for details on the process of capacity-building and mentorship

^b^Programme-supported publications refers to papers supported technically and financially (processing fees in open access journals) by the programme

^c^Includes four papers finalised for publication

### Capacity-building and mentorship for documentation and publication of evidence-based papers

Three cohorts of individuals (hereafter referred to as mentees) were trained over the period 2010–2013. Mentees were primarily programme managers who had not published an evidence-based paper and young academics in the early phase of their careers with no prior experience or formal training in scientific writing. While mentees in the first course were from non-governmental organisations and academic institutions, participants in the second and third courses included programme staff from the government (NACO and State AIDS Control Societies).

**Fig. 1:**
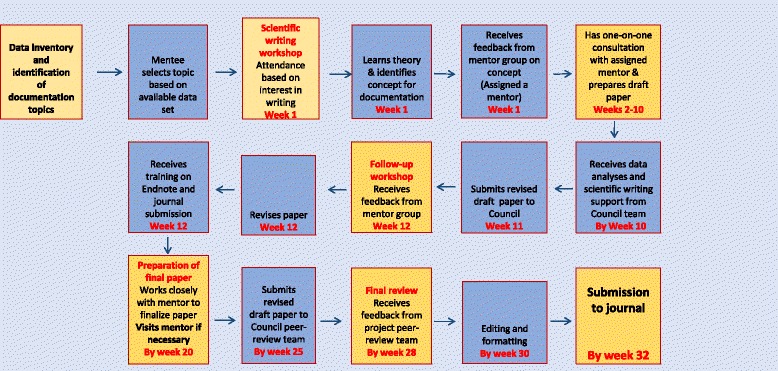
Process of capacity-building and mentorship for documentation and publication of evidence-based papers

The capacity-building and mentorship programme was designed to address the multiple challenges related to the publication of evidence-based papers, such as lack of dedicated time for paper writing, inadequate knowledge of scientific writing conventions, limited skills to analyse and interpret data, limited access to scientific literature, lack of mentorship support and dropout, and psycho-social issues including lack of confidence in writing skills, meeting the publishing standard of peer-reviewed journals, and fear of rejection [1, 12–18]. Workshops were organised to build scientific writing skills and support mentees to conceptualise a topic for documentation based on the programme being implemented, the innovativeness of the programme strategy and the availability of programme data, and follow-up workshops and small group meetings were organised to provide dedicated time for paper writing. Mentorship was an intrinsic component of the capacity-building process, and a group of mentors with diverse skills provided ongoing support and critical feedback to mentees on all aspects of paper writing to take the paper through to publication. The programme followed a structured sequence, with well-defined timelines for preparing and revising papers, and submitting a final paper for publication to a peer-reviewed journal (Fig. 1).

#### Identification of documentation topics

During each scientific writing course, a group of mentors and individual mentees discussed mentees’ areas of interest and the programme area where they were working. Based on the available HIV programme data and identified priority documentation topics to address knowledge gaps, mentees selected a topic of interest for paper writing. The mentors’ group evaluated the uniqueness and innovativeness of the selected topic, following which the analysis team confirmed the availability of reliable data to support the documentation of the topic selected. Through this process, project-supported papers were identified.

Once the documentation topic was selected, mentees identified the type of support needed for analysing and publishing data. No financial incentives were provided for participation in the course or publication.

#### Training in scientific writing

Mentees in each cohort attended a scientific writing workshop for training in skills that are often inadequately developed for writing a peer-reviewed paper, such as scientific writing, topic conceptualisation, data analysis, and interpretation and presentation of results. The first two and a half days of the workshop were assigned to training mentees, through classroom sessions and practical exercises, on the principles of writing a scientific paper and the publishing process based on a module developed by international health experts and customised for research in HIV. Sessions covered the purpose and content of each section of a journal article, and provided a logical formula for scientifically writing each section. The workshop also guided mentees on searching for relevant literature, reviewing the literature, problem conceptualisation, research design and methodology, data analysis, and interpretation and presentation of data.

Additionally, the workshop provided mentees dedicated time to work on conceptualising their paper using a preselected dataset to address their research question, and during the remaining two and a half days, mentees were supported to conceptualise and prepare an extended abstract on their assigned topic. Each mentee was assigned a mentor, based on the kind of support needed and topic being documented, who provided feedback during the workshop as well as follow-up support to guide the paper to completion. Based on individual feedback, as well as collective feedback from the mentors’ group, on the feasibility of documenting the proposed research topic, formulating their research question and conceptualising their research topic, mentees prepared an extended abstract of their paper. Data analysis support was provided as needed. Extended abstracts, with a clear conceptualisation of measures, analysis plan and preliminary results were presented at the end of the workshop for feedback from the mentors’ group. Time-lines for preparing draft and final papers were set at the workshop to ensure mentees’ momentum and interest in paper writing.

#### Follow-up with mentees for preparation of draft papers

Over the next 10 weeks, extended abstracts were reviewed by assigned mentors, and mentees were given detailed feedback on email/Skype. Based on these comments, mentees modified their paper and submitted a first draft for review to their assigned mentor and the Council/project team. At the same time, mentees were provided additional resources, including access to published literature, data, programme content, and data analysis and scientific writing support, to enable them to complete a draft paper.

#### Follow-up workshop to revise draft papers

As most mentees were involved with routine work once they returned to office, with limited time for paper writing, a follow-up five-day offsite workshop was organised to provide mentees dedicated time to review and revise their paper and prepare a near-final draft for submission to a peer-reviewed journal. With guidance from assigned mentors, and data analysis and scientific writing support, mentees revised their papers, which were shared with the mentor group for further review. Following a review of the comments by the assigned mentor, mentees prepared a near-final draft paper. Additionally, mentees were trained on the use of Endnote (a web-based reference manager), guided on the journal submission and review process, and provided a comprehensive journal information guide (including areas of interest, contact information, instructions for authors and impact factor) to help them select an appropriate journal for publication. At the end of the workshop, mentees presented an overview of their revised paper to the mentor group for another round of feedback and review.

#### Preparation of final papers

Mentees worked closely with their assigned mentors and the Council team after the second workshop (weeks 13–24) to further revise their papers and prepare a final draft. Where necessary, the Council/project team organised small group meetings to facilitate mentor-mentee discussions, and provide data analysis and writing support for paper finalisation.

#### Final review and submission of papers to a journal for publication

Following multiple rounds of feedback and revision, mentees submitted a final draft paper to the Council peer-review team (weeks 25–32). Based on their feedback, mentees finalised their paper. The final papers were technically edited, formatted according the selected journal style and submitted to the journal for publication. If necessary, mentees were supported through the submission process.

### Measures

The following measures were used to assess the overall performance and impact of project publications. We used Google Scholar to retrieve paper-specific citation metrics for programme published papers, including the total number of citations, citations per paper and the i10 index (number of papers with at least 10 citations). Additionally, the impact factor of the journal in which each paper was published (2015/2016 Thompson Reuters ranking or the impact factor indicated on the journal home page) was used as a proxy indicator for quality. We also assessed the number of downloads/views for each paper based on information drawn from the journal site. Data for this paper cover the period January 2010 to December 2016.

### Data analysis

Data for the 99 project-supported papers were entered in MS Excel and analysed. Results are presented as absolute numbers or percentages relative to the base indicator.

The scientific writing and follow-up workshops were evaluated based on mentees’ feedback. Following each workshop, mentees were asked to fill in an anonymous evaluation questionnaire, including rating their improvement in knowledge following participation in the workshop, and the likelihood of using the information and skills gained in their future work. Responses ranged from 0 (not at all) to 2 (very much). Open-ended questions were included on what mentees liked best/least about the course, which components could be changed and recommendations to improve effectiveness. Responses to open-ended questions in the course evaluation questionnaires are presented as verbatim quotes. Participants have been given fictitious names to ensure confidentiality.

Published papers were thematically reviewed and classified into nine broad themes, and across multiple categories, to identify themes where papers exhibited the greatest impact. Themes included the key populations’ (female sex workers, high-risk men who have sex with men, transgender persons and injecting drug users) HIV/sexually transmitted infection (STI) risk, HIV/STI risk of general population groups, bridge populations’ (truckers, clients of female sex workers and male migrants) HIV/STI risk, HIV prevention programmes, HIV/STI service delivery models (e.g. integration of HIV and maternal health services, and public–private provider care models), community mobilisation interventions, impact of scaled-up HIV prevention programmes, monitoring and evaluation methodologies, and behaviour change models.

### Ethics statement

Ethical approval for the study was submitted to the Population Council’s Institutional Review Board, which exempted the project from review as it did not involve any primary data collection.

## Results

### Peer-reviewed evidence-based papers published under the programme

A total of 32% (99/312) of the papers on the HIV epidemic in India published in peer-reviewed journals (2006–2016) are outputs of the programme (Table 2). Of these, 95 programme-supported papers have been published in national and international peer-reviewed journals (Additional file 1). On average, 14 programme-supported papers were published each year. Almost three-quarters of these published papers (72%; 68/95) have been cited at least once in the literature.

**Table 2:** Papers published on the HIV epidemic in India by year, 2006–2016: Number published by all HIV programmes, number published under the project (programme-supported), and number of published programme-supported papers cross-referenced

Year	No. of papers published from all HIV programmes in India^a^	No. of programme-supported papers^b^ published	No. of program-supported papers^b^ cross-referenced in the literature^d^
2006–2010	65	5	5
2011	37	8	8
2012	58	18	16
2013	45	16	14
2014	51	21	18
2015	39	21^c^	5
2016	17	10	2
Total	312	99	68

^a^Includes 99 programme-supported papers

^b^Refers to papers supported technically and financially (for processing fees in open access journals) by the programme

^c^Includes 4 papers finalised for publication

^d^Includes 95 published papers cross-referenced at least once

### Publication output from the capacity-building and mentorship programme

The capacity-building and mentorship programme, which provided mentees ongoing guidance and support for documentation and publication of evidence-based papers, resulted in a significant number of publications. Overall, 70 mentees were trained and supported over the programme, and 10 NDAP analysts were mentored by the programme to finalise papers. Over two-thirds (68%; 67/99) of the papers finalised under the Knowledge Network project have been co-authored by mentees.

The capacity-building and mentorship process also resulted in several individual-level benefits. Mentees reported enhanced documentation skills, including making them more adept in writing different sections of an evidence-based paper, understanding the process of conceptualising a research question and interpreting and analysing programme data to understand results on the ground. It also created an appreciation of the importance of a well-constructed abstract, selection of appropriate journals for paper submission and an understanding of the process of submission and correspondence with journal editors and reviewers.

*“Interaction with resource persons and practice exercises greatly helped to clarify doubts*.” Sheila, workshop participant

*“The paper concept is clear to us now. This course helped us to learn many things, including how we should look at data, how it should be interpreted and how to measure programme effectiveness.”* Ram, workshop participant

As a result of ongoing support, motivation and encouragement, mentees developed confidence in their ability to document HIV programme lessons and publish evidence-based papers. The process also created an appreciation of the need for data to assess programme impact.

*“Mentors gave valuable inputs and created a supportive environment, which improved our confidence.”* Rita, workshop participant

*“This has been one of the most interesting workshops; for those who do not come from a research background, being part of the workshop provided confidence in the programme implementation strategy* [and helped us realise that] *writing a scientific paper is possible… we need to see the result of the programme on the ground* [and this helped us] *see the impact of programmes.... Support was good.*” Vinod, workshop participant

### Contribution to the literature on the HIV epidemic in India

**Fig. 2:**
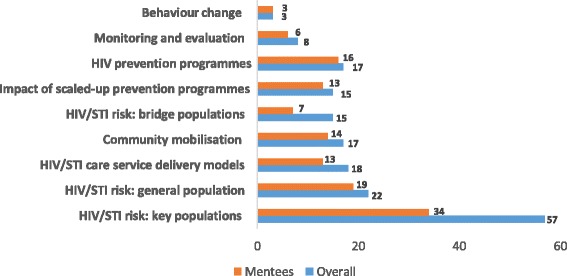
Number of papers published under the project by theme: Overall and by mentees, 2010–2016

The programme contributed to building the evidence on multiple themes on the HIV epidemic in India. The 99 programme-supported papers are primarily original research articles, 2 are systematic reviews and 3 are case studies. Overall, of the published papers (95), 60% covered issues related to key populations’ HIV risk, 23% documented the HIV risk of general population groups, 19% documented HIV/STI service delivery models and 18% documented community mobilisation interventions (Fig. 2). Most papers co-authored by mentees documented key populations’ HIV risk (54%), followed by the risk of general population groups (30%), HIV prevention programmes (25%) and community mobilisation interventions (22%).

### Journal impact factor and citation rates

Programme-supported papers have been published in 38 different journals, of which 19 have been published in journals with an impact factor of 1–1.99, 14 in journals with an impact factor of 2–2.99, and 25 in journals with an impact factor of 3.0 or more (not shown in tabular form).

Results from the Google Scholar citation index indicate that the 95 programme-supported published papers have been cited 952 times; 72% (68/95) have been cited at least once and 33% have been cited at least 10 times (i10 index). Further, of the 95 published papers, 79% (75/95) are included in the Scopus/PubMed/Web of Science/IndMED databases. Citations over the period increased from 31 in 2011 to 214 in 2016 (not shown in tabular form).

In terms of thematic impact (Table 3), on average, each paper has been cited 10.4 times, with above-average citations of papers documenting key populations’ risk, bridge populations’ risk, HIV care service delivery models, and monitoring and evaluation methodologies. The largest number of average downloads/views of papers covered the themes of HIV/STI service delivery, community mobilisation interventions, impact assessments of programme effectiveness and HIV prevention programmes. Papers published by mentees (63) were cited on average 5.4 times, with above-average citations of papers relating to HIV risk of key populations, HIV care service delivery models and community mobilisation interventions. Behaviour change models were less frequently documented and cited in the literature.

**Table 3:** Programme-supported papers published overall and papers published by mentees, 2010–2016: Citations and downloads by theme

Theme documented	All programme-supported papers^a^ published^b^			Programme-supported papers^a^ published by mentees^c^	
	No. of citations (No. of papers)	Average citations per paper	Average downloads/views per paper	No .of citations (No. of papers)	Average citations per paper
HIV/STI risk: key populations^d^	608 (57)	10.7	1656	230 (34)	6.8
HIV/STI risk: bridge populations^e^	298 (15)	19.9	1551	41 (7)	5.9
HIV/STI care service delivery models	248 (18)	13.8	2091	85 (13)	6.5
Impact of scaled-up prevention programmes	77 (15)	5.1	1823	44 (13)	3.4
Community mobilisation interventions	146 (17)	8.6	1985	89 (14)	6.4
HIV prevention programmes	150 (17)	8.8	1644	69 (16)	4.3
HIV/STI risk: general population	134 (22)	6.1	1362	80 (19)	4.2
Monitoring and evaluation methodologies	111 (8)	13.9	1354	23 (6)	3.8
Behaviour change models	11 (3)	3.7	407	11 (3)	3.7
Total	1796 (172)	10.4	1656	672 (125)	5.4

Note: Based on Google Scholar citation list. Papers are grouped into multiple thematic categories

^a^Refers to papers supported technically and financially (for processing fees in open access journals) by the programme

^b^Includes 95 published papers

^c^Includes 63 published papers

^d^Includes female sex workers, high-risk men who have sex with men, transgender persons and injecting drug users

^e^Includes truckers, clients of female sex workers and migrants

## Discussion

This study demonstrates that HIV programme data, covering a range of geographies, time periods, population groups and intervention strategies, can be synthesised and programmatic lessons published as evidence-based papers in peer-reviewed journals. The Knowledge Network project was initiated during NACP III (2007–2012), when the HIV prevention programme was being scaled up across the country and the need for an evidence-informed policy formulation process was recognised [4], yet research output from India was limited [9]. Through the adoption of an integrated approach, including the publication of papers on identified priority documentation topics in partnership with a core group of academics, and training and mentoring HIV prevention programme managers and young academics to publish peer-reviewed papers, the programme contributed significantly to the overall literature on the HIV epidemic in India and, to date, the project has supported the finalisation and publication 99 peer-reviewed papers on HIV programmatic learnings (Additional file 1). The capacity-building programme chartered new ground by training and mentoring participants, most of whom had no prior publishing experience, to document and publish programmatic lessons from the HIV epidemic in India, and as a result, over two-thirds of the overall programme publications are co-authored by programme-supported mentees.

The publication rate under the programme – 99 papers over the project period – far exceeded the output envisioned under the project (10 publications a year). The high publication rates from the capacity-building programme can be attributed to several factors. For one, the training course was sequenced, with well-coordinated activities and clearly identified goals, which kept mentees motivated and focused on timelines and deliverables [14]. Second, the programme brought together individuals with programme experience (mentees) and mentors with scientific rigor from academic and research institutions in a symbiotic partnership to build sound evidence from the data; while mentors provided mentees ongoing support throughout the topic conceptualisation, analysis and writing phases, programme managers who are ‘data producers and users’ provided insights from the data to inform appropriate programme strategies and priorities on the ground. As documented elsewhere, ongoing mentorship is critical to achieve high publication outcomes, and reduce drop-out and delay [1, 13, 18, 19]. Moreover, mentor-mentee matching – where each mentee was paired with a mentor with expertise in their field – optimised interaction and guidance, and facilitated the timely completion of quality papers [15]. Third, regular monitoring by the project team ensured the quality and progress of work. In cases of delays, small group meetings were organised with mentors and resource persons to enable mentees to discuss problems and seek support to complete their paper. Fourth, resource persons provided mentees with data analysis and scientific writing support, and access to published literature and other resources, as needed. The careful selection of mentees based on their interest and commitment ensured most were able to complete a peer-reviewed paper.

Supporting mentees to analyse their own datasets and publish the lessons helped to build their skills in documentation, develop confidence in their ability to achieve high publication standards, and created an appreciation of the need for collecting quality data and the benefits of robust research, which can be translated into scientific evidence. However, despite intensive efforts, some mentees were unable to submit a final paper to a journal for publication due to job transition or burnout; in such cases, for the most part, other participants from the same institution were able to complete the assigned paper. Mentors were sometimes unable to commit dedicated time for ongoing mentorship. Regular follow-up and monitoring of deliverables helped to ensure that activities stayed on track and reduce burnout. As a result, most mentees achieved their goal of co-authoring an evidence-based paper on HIV programme lessons for publication in a peer-reviewed journal, while some mentees co-authored two or more papers over the project period.

The programme built a sound evidence base on the HIV epidemic in India through the publication of a number of peer-reviewed papers. Most papers were published in high impact journals, indicating that the evidence is being disseminated to a wide audience, the output is high quality and meets the rigorous standards of peer-reviewed publications. These papers drew on multiple datasets, including programme evaluation data, bio-behavioural surveys, mapping exercises and secondary data to document a range of programme-related themes, presenting a comprehensive picture of the HIV epidemic in India. These publications have been widely cited in the literature; multiple citations indicate that the papers are widely recognised by researchers in the field and are being linked with prior work in the scientific literature. As discussed, research topics were carefully selected to address knowledge gaps and to align with national programme priorities, which may have contributed to high citation rates. Moreover, several themes documented under the project and which were highly cited, including the impact of community mobilisation interventions, the HIV risk of general population groups (antenatal clinic attendees, blood donors, slum populations, married women, and adult men and women) and bridge populations (long-distance truckers and clients of female sex workers), and HIV service delivery interventions (peer-led outreach, sex worker-led crisis response systems, integration of HIV and maternal health services, and the provision of anti-retroviral treatment services), reflect a shift in the focus of HIV prevention research in India. Issues such as the HIV vulnerability of mobile populations in India, primarily male migrant workers, have been extensively documented for perhaps the first time under the programme. Notably, mentees, who were mainly programme managers with a sound understanding of issues on the ground, and able to identify appropriate needs and suggest relevant ground-level programmatic solutions, made a substantial contribution to the literature on several themes such as key populations’ risk, bridge populations’ risk, community mobilisation interventions and lessons from HIV prevention programmes. Our study suggests that efforts are needed to build the evidence on less frequently documented and cited themes such as behaviour change models for HIV prevention.

The programme also highlights the value of peer-reviewed publications in shaping policy. NACO has incorporated lessons from the published literature on migrants’ HIV risk into the national programme, and India has adopted a national HIV prevention strategy that focuses on corridors of migration, which include hometowns, destinations and the transit points between them, rather than destination areas alone.

High publication output, multiple citations and publications in high-impact factor journals together suggest that the programme has been successful in building a robust evidence base on the HIV epidemic, contributing significantly to the overall published literature on the HIV epidemic in India and informing the national prevention programme in a relatively short time span of 6 years. These results suggest that the capacity-building and mentorship model described in this paper can be applied in different settings to build scientific writing and documentation capacity in other public health programmes that are implemented at scale.

The successful implementation of the programme had several spinoffs. While the programme initially focused on supporting programme implementers from non-governmental organisations to document and publish lessons from their programme data, efforts were scaled up to support participants from NACO and State AIDS Control Societies to analyse their HIV programme data and publish the evidence in peer-reviewed journals. Moreover, NACO adopted a similar capacity-building and mentorship model in 2014–2015 to support the analysis of national HIV/AIDS programme data under NDAP. The Knowledge Network project partnered with NACO to build the documentation capacity of over 40 NDAP analysts through scientific writing workshops and supported analysts to finalise and publish programme data as peer-reviewed papers. The programme also built an appreciation of the need to strengthen research capacity to support the documentation and publication of HIV programme data in India and to develop the documentation skills of future researchers. Following requests from government and academic institutes (MSACS, Family Welfare Resource and Training Center and Tata Institute of Social Sciences), scientific writing workshops were organised.

While this study provides insights into a novel initiative for the documentation and publication of HIV programmatic learnings from India, the findings need to be interpreted in light of certain limitations. First, citation analysis can only be applied to published literature in journals that are indexed; however, some programme papers may have been published in non-indexed journals and may therefore not have been included in the analysis. Further, citations are treated as equal irrespective of whether the work is being cited for its positive contribution or critiqued for its poor quality. In addition, the citation figures for more recent years are lower because papers published during this period have had less time to accumulate citations. Moreover, given the brief assessment period, some papers may not have established their presence in the publication domain, and therefore our analysis may not have captured the full variability of citation patterns of a mature set of publications. We have reported the journal impact factor for 2015 or 2016; however, a journal’s impact factor changes from year to year. Moreover, caution must be used in emphasising the scientific quality of publication output based solely on citation and impact factor metrics due to the potential to generate misleading and biased results [20]. Additionally, the effect of the capacity-building programme was self-reported, and the possibility of respondent bias needs to be considered.

## Conclusion

This study demonstrates that the adoption of an integrated approach can support the documentation and publication of programmatic lessons as evidence-based papers in peer-reviewed journals. As seen in this study, the programme contributed significantly to the overall published literature on the HIV epidemic in India and informed HIV prevention programmes and policy in the country. As documented, a well-structured and integrated documentation programme can result in a high publication output, multiple citations and publications in high-impact factor journals. The capacity-building model described in this paper, which set well-defined targets for deliverables, ensured ongoing mentorship and provided regular monitoring, can be applied to build scientific writing and documentation capacity in other public health programmes that are implemented at scale.

## Additional file

Additional file 1: Papers on the HIV epidemic in India published under the programme. (DOCX 37 kb)

## Abbreviations

MSACS: Maharashtra State AIDS Control Society
NACO: National AIDS Control Organisation
NACP: National AIDS Control Programme
NDAP: National Data Analysis Plan
STI: Sexually transmitted infection

## References

[1] Mugabo L, Rouleau D, Odhiambo J, Nisingizwe MP, Amoroso C, Barebwanuwe P, Warugaba C, Habumugisha L, Hedt-Gauthier BL (2015). Approaches and impact of non-academic research capacity strengthening training models in sub-Saharan Africa: a systematic review. Health Res Policy Syst.

[2] National AIDS Control Organisation (NACO) (2014). Annual Report 2012–13.

[3] National AIDS Control Organization (NACO) (2010). National AIDS Control Program: Response to the HIV Epidemic in India.

[4] Tran NT, Bennett SC, Bishnu R, Singh S (2013). Analyzing the sources and nature of influence: how the Avahan program used evidence to influence HIV/AIDS prevention policy in India. Implement Sci.

[5] Dandona L (2004). Enhancing the evidence base for HIV/AIDS control in India. Natl Med J India.

[6] Dandona L, Sivan YS, Jyothi MN, Bhaskar VS, Dandona R (2004). The lack of public health research output from India. BMC Public Health.

[7] Paraje G, Sadana R, Karam G (2005). Public health. Increasing international gaps in health-related publications. Science.

[8] Dandona L, Raban MZ, Guggilla RK, Bhatnagar A, Dandona R (2009). Trends of public health research output from India during 2001-2008. BMC Med.

[9] Chand P (2007). HIV/AIDS research in India: A bibliometric study. Libr Inf Sci Res.

[10] Verma R, Shekhar A, Khobragade S, Adhikary R, George B, Ramesh BM, Ranebennur V, Mondal S, Patra RK, Srinivasan S (2010). Scale-up and coverage of Avahan: a large-scale HIV-prevention programme among female sex workers and men who have sex with men in four Indian states. Sex Transm Infect.

[11] Bill & Melinda Gates Foundation (2008). Avahan – The India AIDS Initiative: The Business of HIV Prevention at Scale.

[12] Nchinda TC (2002). Research capacity strengthening in the South. Soc Sci Med.

[13] Pager S, Holden L, Golenko X (2012). Motivators, enablers, and barriers to building allied health research capacity. J Multidiscip Healthc.

[14] McGrail MR, Rickard CM, Jones R (2006). Publish or perish: a systematic review of interventions to increase academic publication rates. High Educ Res Dev.

[15] Chu KM, Jayaraman S, Kyamanywa P, Ntakiyiruta G (2014). Building research capacity in Africa: equity and global health collaborations. PLoS Med.

[16] Holstein SE, Mickley Steinmetz KR, Miles JD (2015). Teaching science writing in an introductory lab course. J Undergrad Neurosci Educ.

[17] Freeman P, Robbins A (2006). The publishing gap between rich and poor: the focus of AuthorAID. J Public Health Policy.

[18] Lansang MA, Dennis R (2004). Building capacity in health research in the developing world. Bull World Health Organ.

[19] Thomson DR, Semakula M, Hirschhorn LR, Murray M, Ndahindwa V, Manzi A, Mukabutera A, Karema C, Condo J, Hedt-Gauthier B (2016). Applied statistical training to strengthen analysis and health research capacity in Rwanda. Health Res Policy Syst.

[20] Pendlebury DA (2009). The use and misuse of journal metrics and other citation indicators. Arch Immunol Ther Exp.

