# Supplementary material for: Building the evidence base on the HIV programme in India: an integrated approach to document programmatic learnings
Source: Health Res Policy Syst. 2018 Mar 12;16:22. doi: 10.1186/s12961-018-0291-3 (PMC5848569; doi:10.1186/s12961-018-0291-3)
Supplement: Supplementary file 1 — Papers on the HIV epidemic in India published under the programme. (DOCX 37 kb) [file 12961_2018_291_MOESM1_ESM.docx]

**Additional file 1:** Papers on the HIV epidemic in India published under the programme

Papers on the HIV epidemic in India published under the programme

| 1. | Bharat S, Mahapatra B, Roy S, Saggurti N. Are female sex workers able to negotiate condom use with male clients? The case of mobile FSWs in four high HIV prevalence states of India. PLoS One. 2013;8(6):e68043. http://journals.plos.org/plosone/article/file?id=10.1371/journal.pone.0068043&type=printable |
| --- | --- |
| 2. | Bhardwaj S, Ramesh S, Gaikwad S. Influence of customised demand generation as a strategy to increase uptake of VCT services amongst sex workers in Mumbai and Thane. World J AIDS. 2014;4:139–47. https://file.scirp.org/pdf/WJA_2014061310024311.pdf |
| 3. | Bhattacharjya C, Patel S, Panda S, Deb A. Changing HIV epidemic in North-Eastern India and its relationship with development and programmatic indicators. World J AIDS. 2015;5:265–74. https://file.scirp.org/pdf/WJA_2015092514502749.pdf |
| 4. | Bhattacharjya C, Sahu D, Patel SK, Saggurti N, Pandey A. Causes of death among HIV-infected adults registered in selected anti-retroviral therapy centers in north-eastern India. World J AIDS. 2015;5:90–9. https://file.scirp.org/pdf/WJA_2015060314070335.pdf |
| 5. | Bindoria SV, Devkar R, Gupta I, Ranebennur V, Saggurti N, Ramesh S, Deshmukh D, Gaikwad S. Development and pilot testing of HIV screening program integration within public/primary health centers providing antenatal care services in Maharashtra, India. BMC Res Notes. 2014;7:177. https://www.ncbi.nlm.nih.gov/pmc/articles/PMC3986915/pdf/1756-0500-7-177.pdf |
| 6. | Chakravarthy JB, Joseph SV, Pelto P, Kovvali D. Community mobilisation programme for female sex workers in coastal Andhra Pradesh, India: processes and their effects. J Epidemiol Community Health. 2012;66 Suppl 2:ii78–86. http://jech.bmj.com/content/jech/66/Suppl_2/ii78.full.pdf |
| 7. | Choudhury L, Prabakaran J. Urban and rural HIV Estimates among adult population (15–49 years) in selected states of India using spectrum data. World J AIDS. 2015;5:226–37. https://file.scirp.org/pdf/WJA_2015092513540282.pdf |
| 8. | Deshpande S, Bharat S. Sexual partner mixing and differentials in consistent condom use among men who have sex with men in Maharashtra, India. Glob Public Health. 2015;10(1):103–18. http://www.tandfonline.com/doi/abs/10.1080/17441692.2014.972968?journalCode=rgph20 |
| 9. | Gaikwad SS, Bhende A, Nidhi G, Saggurti N, Ranebennur V. How effective is community mobilisation in HIV prevention among highly diverse sex workers in urban settings? The Aastha intervention experience in Mumbai and Thane districts, India. J Epidemiol Community Health. 2012;66 Suppl 2:ii69–77. http://jech.bmj.com/content/jech/66/Suppl_2/ii69.full.pdf |
| 10. | Galavotti C, Wheeler T, Kuhlmann AS, Saggurti N, Narayanan P, Kiran U, Dallabetta G. Navigating the swampy lowland: a framework for evaluating the effect of community mobilisation in female sex workers in Avahan, the India AIDS Initiative. J Epidemiol Community Health. 2012;66 Suppl 2:ii9–15. http://jech.bmj.com/content/jech/early/2012/07/01/jech-2011-200465.full.pdf |
| 11. | Ganju D, Mahapatra B, Saggurti N. Male migrants’ non-spousal sexual partnerships in the place of origin: an in-depth investigation in two rural settings of India. Culture Health Sex. 2013;15(3):341–57. http://www.tandfonline.com/doi/pdf/10.1080/13691058.2012.756932?needAccess=true |
| 12. | Ganju D, Patel SK, Prabhakar P, Adhikary R. Knowledge and exercise of human rights, and barriers and facilitators to claiming rights: a cross-sectional study of female sex workers and high-risk men who have sex with men in Andhra Pradesh, India. BMC Int Health Human Rights. 2016;16:29. https://www.ncbi.nlm.nih.gov/pmc/articles/PMC5112884/pdf/12914_2016_Article_102.pdf |
| 13. | Ganju D, Ramesh S, Saggurti N. Factors associated with HIV testing among male injecting drug users: findings from a cross-sectional behavioural and biological survey in Manipur and Nagaland, India. Harm Reduct J. 2016;13:21. https://www.ncbi.nlm.nih.gov/pmc/articles/PMC4915098/pdf/12954_2016_Article_110.pdf |
| 14. | Ganju D, Saggurti N. Stigma, violence and HIV vulnerability among transgender persons in sex work in Maharashtra, India. Cult Health Sex. 2017;19(8):903–17. http://www.tandfonline.com/doi/pdf/10.1080/13691058.2016.1271141?needAccess=true |
| 15. | Gupta I, Joe W, Rudra S. HIV prevention: Towards a ‘structural plus’ approach. Health Aff (Millwood). 2013;5:102–8. http://file.scirp.org/pdf/Health_2013011808534709.pdf |
| 16. | Gurav K, Bradley J, Chandrashekhar Gowda G, Alary M. Perspectives on condom breakage: a qualitative study of female sex workers in Bangalore, India. Culture Health Sex. 2014;16(5):575–86. http://www.tandfonline.com/doi/abs/10.1080/13691058.2014.883642?journalCode=tchs20 |
| 17. | Halli SS, Buzdugan R, Moses S, Blanchard J, Jain A, Verma R, Saggurti N. High-risk sex among mobile female sex workers in the context of jatras (religious festivals) in Karnataka, India. Int J STD AIDS. 2010;21(11):746–51. https://www.ncbi.nlm.nih.gov/pubmed/21187355 |
| 18. | Jain AK, Saggurti N. The extent and nature of fluidity in typologies of female sex work in Southern India: implications for HIV prevention programs. J HIV AIDS Soc Serv. 2012;11(2):169–91. https://www.ncbi.nlm.nih.gov/pmc/articles/PMC3379737/pdf/whiv11_169.pdf |
| 19. | Jain AK, Saggurti N, Mahapatra B, Sebastian MP, Modugu HR, Halli SS, Verma RK. Relationship between reported prior condom use and current self-perceived risk of acquiring HIV among mobile female sex workers in southern India. BMC Public Health. 2011;11 Suppl 6:S5. https://www.ncbi.nlm.nih.gov/pmc/articles/PMC3287558/pdf/1471-2458-11-S6-S5.pdf |
| 20. | Jain B, Krishnan S, Ramesh S, Sabarwal S, Garg V, Dhingra N. Effect of peer-led outreach activities on injecting risk behavior among male drug users in Haryana, India. Harm Reduct J. 2014;11:3. https://www.ncbi.nlm.nih.gov/pmc/articles/PMC3922610/pdf/1477-7517-11-3.pdf |
| 21. | Jha PK, Sahu D, Srikanth Reddy K, Narayan P, Pandey A. Multiple sexual partners and vulnerability to HIV: a study of patterns of sexual behaviour in the slum population of India. World J AIDS. 2014;4:373–81. http://file.scirp.org/pdf/WJA_2014111413073006.pdf |
| 22. | Jha PK, Narayan P, Nair S, Ganju D, Sahu D, Pandey A. An assessment of comprehensive knowledge of HIV/AIDS among slum and non-slum populations in Delhi, India. Open J Prev Med. 2015;5:259–68. http://file.scirp.org/pdf/OJPM_2015061913550984.pdf |
| 23. | Jha UM, Raj Y, Venkatesh S, Dhingra N, Paranjpe RS, Saggurti N. HIV epidemic among men who have sex with men in India: national scenario of an unfinished agenda. HIV AIDS. 2014;6:159–70. https://www.ncbi.nlm.nih.gov/pmc/articles/PMC4242134/pdf/hiv-6-159.pdf |
| 24. | Juneja S, Rao TV, Mishra RM, Sethu S, Singh IR. Impact of an HIV prevention intervention on condom use among long distance truckers in India. AIDS Behav. 2013;17(3):1040–51. https://www.ncbi.nlm.nih.gov/pmc/articles/PMC3586141/pdf/10461_2012_Article_314.pdf |
| 25. | Kokku SB, Mahapatra B, Tucker S, Saggurti N, Prabhakar P. Effect of public-private partnership in treatment of sexually transmitted infections among female sex workers in Andhra Pradesh, India. Indian J Med Res. 2014;139(2):285–93. https://www.ncbi.nlm.nih.gov/pmc/articles/PMC4001342/?report=printable |
| 26. | Kuhlmann AS, Galavotti C, Hastings P, Narayanan P, Saggurti N. Investing in communities: evaluating the added value of community mobilization on HIV prevention outcomes among FSWs in India. AIDS Behav. 2014;18(4):752–66. https://www.ncbi.nlm.nih.gov/pmc/articles/PMC3961594/pdf/10461_2013_Article_626.pdf |
| 27. | Mahapatra B, Saggurti N, Halli SS, Jain AK. HIV risk behaviors among female sex workers using cell phone for client solicitation in India. J AIDS Clin Res. 2012;S1:014. file:///C:/Users/DF/Downloads/hiv-risk-behaviors-among-female-sex-workers-using-cell-phone-for-client-solicitation-in-india-2155-6113.S1–014.pdf |
| 28. | Mahapatra B, Battala M, Porwal A, Saggurti N. Non-disclosure of violence among female sex workers: evidence from a large scale cross-sectional survey in India. PLoS One. 2014;9(5):e98321. http://journals.plos.org/plosone/article/file?id=10.1371/journal.pone.0098321&type=printable |
| 29. | Mahapatra B, Lowndes CM, Gurav K, Ramesh BM, Moses S, Washington R, Alary M. Degree and correlates of sexual mixing in female sex workers in Karnataka, India. Sex Health. 2013;10(4):305–10. http://www.publish.csiro.au/sh/pdf/SH12215 |
| 30. | Mahapatra B, Lowndes CM, Mohanty SK, Gurav K, Ramesh BM, Moses S, Washington R, Alary M. Factors associated with risky sexual practices among female sex workers in Karnataka, India. PLoS One. 2013;8(4):e62167. http://journals.plos.org/plosone/article/file?id=10.1371/journal.pone.0062167&type=printable |
| 31. | Mahapatra B, Saggurti N. Exposure to pornographic videos and its effect on HIV-related sexual risk behaviours among male migrant workers in southern India. PLoS One. 2014;9(11):e113599. http://journals.plos.org/plosone/article/file?id=10.1371/journal.pone.0113599&type=printable |
| 32. | Mishra RK, Ganju D, Ramesh S, Lalmuanpuii M, Biangtung L, Humtsoe C, Saggurti N. HIV risk behaviors of male injecting drug users and associated non-condom use with regular female sexual partners in north-east India. Harm Reduct J. 2014;11:5. https://www.ncbi.nlm.nih.gov/pmc/articles/PMC3932044/pdf/1477-7517-11-5.pdf |
| 33. | Mishra RM, Dube M, Saggurti N, Pandey A, Mahapatra B, Ramesh S. The association between adolescent entry into the trucking industry and risk of HIV among long-distance truck drivers in India. HIV AIDS. 2012;4:141–8. https://www.ncbi.nlm.nih.gov/pmc/articles/PMC3435090/pdf/hiv-4-141.pdf |
| 34. | Mishra RM, Dube M, Sahu D, Saggurti N, Pandey A. Changing epidemiology of HIV in Mumbai: an application of the Asian epidemic model. Glob J Health Sci. 2012;4(5):100–12. https://www.ncbi.nlm.nih.gov/pmc/articles/PMC4776940/pdf/GJHS-4-100.pdf |
| 35. | Mondal S, Gupta I. How costly is safe sex? An economic analysis of the sex market in India. Global Health Perspect. 2013;1:25–32. www.researchgate.net/publication/314546547_How_Costly_Is_Safe_Sex_An_Economic_Analysis_of_the_Commercial_Sex_Market_in_India |
| 36. | Nagarajan K, Godbole S, Ramakrishnan L, Mainkar MK, Ramesh S, Ganju D, Paranjape RS. Self-report of STI symptoms, inconsistent condom use and condom non-use are poor predictors of STI prevalence among men who have sex with men. J AIDS Clin Res. 2013;4:212. ile:///C:/Users/DF/Downloads/self-report-of-sti-symptoms-inconsistent-condom-use-and-condom-nonuse-are-poor-predictors-of-sti-prevalence-among-men-who-have-sex-with-men-2155-6113.1000212.pdf |
| 37. | Nagarajan K, Sahay S, Ganju D, Paranjape RS. Public participation of men who have sex with men in the context of community empowerment in India. J AIDS Clin Res. 2015;6:509. file:///C:/Users/DF/Downloads/public-participation-of-men-who-have-sex-with-men-in-the-context-ofcommunity-empowerment-in-india-2155-6113-1,000,509.pdf |
| 38. | Nagarajan K, Sahay S, Mainkar MK, Deshpande S, Ramesh S, Paranjape RS. Female sex worker’s participation in the community mobilization process: two distinct forms of participations and associated contextual factors. BMC Public Health. 2014;14:1323. https://bmcpublichealth.biomedcentral.com/track/pdf/10.1186/1471-2458-14-1323?site=bmcpublichealth.biomedcentral.com |
| 39. | Narayanan P, Moulasha K, Wheeler T, Baer J, Bharadwaj S, Ramanathan TV, Thomas T. Monitoring community mobilisation and organisational capacity among high-risk groups in a large-scale HIV prevention programme in India: selected findings using a Community Ownership and Preparedness Index. J Epidemiol Commun Health. 2012;66 Suppl 2:ii34–41. http://jech.bmj.com/content/jech/66/Suppl_2/ii34.full.pdf |
| 40. | Nuken A, Kermode M, Saggurti N, Armstrong G, Medhi GK. Alcohol and condom use among HIV-positive and HIV-negative female sex workers in Nagaland, India. Int J STD AIDS. 2013;24(9):695–702. https://www.ncbi.nlm.nih.gov/pubmed/23970581 |
| 41. | Pandey A, Mishra RM, Sahu D, Benara SK, Biswas M, Sengupta U, Mainkar MK, Adhikary R. Heterosexual risk behavior among long distance truck drivers in India: role of marital status. Indian J Med Res. 2012;136(supplement):44–53. http://icmr.nic.in/ijmr/2012/october%20supp/7.pdf |
| 42. | Pandey A, Benara SK, Roy N, Sahu D, Thomas M, Joshi DK, Sengupta U, Paranjape RS, Bhalla A, Prakash A. Risk behaviour, sexually transmitted infections and HIV among long-distance truck drivers: a cross-sectional survey along national highways in India. AIDS. 2008;22 Suppl 5:S81–90. https://www.ncbi.nlm.nih.gov/pubmed/19098482 |
| 43. | Pandey A, Mishra RM, Reddy DC, Thomas M, Sahu D, Bharadwaj D. Alcohol use and STI among men in India: evidences from a national household survey. Indian J Commun Med. 2012;37(2):95–100. https://www.ncbi.nlm.nih.gov/pmc/articles/PMC3361808/?report=printable |
| 44. | Pandey A, Mishra RM, Sahu D, Benara SK, Sengupta U, Paranjape RS, Gautam A, Lenka SR, Adhikary R. Heading towards the Safer Highways: an assessment of the Avahan prevention programme among long distance truck drivers in India. BMC Public Health. 2011;11 Suppl 6:S15. https://www.ncbi.nlm.nih.gov/pmc/articles/PMC3287553/pdf/1471-2458-11-S6-S15.pdf |
| 45. | Parimi P, Mishra RM, Tucker S, Saggurti N. Mobilising community collectivisation among female sex workers to promote STI service utilisation from the government healthcare system in Andhra Pradesh, India. J Epidemiol Community Health. 2012;66(Suppl 2):ii62–8. http://jech.bmj.com/content/jech/66/Suppl_2/ii62.full.pdf |
| 46. | Patel SK, Batalla M, Adhikary R. Food insufficiency, violence and HIV risk behaviors among female sex workers in India. J Food Security. 2016;4(5):104–11. file:///C:/Users/DF/Downloads/jfs-4-5-1%20(1).pdf |
| 47. | Patel SK, Ganju D, Prabhakar P, Adhikary R. Relationship between mobility, violence and major depression among female sex workers: a cross-sectional study in southern India. BMJ Open. 2016;6(9):e011439. http://bmjopen.bmj.com/content/bmjopen/6/9/e011439.full.pdf |
| 48. | Patel SK, Prabhakar P, Jain AK, Saggurti N, Adhikary R. Relationship between community collectivization and financial vulnerability of female sex workers in Southern India. PLoS One. 2016;11(5):e0156060. http://journals.plos.org/plosone/article/file?id=10.1371/journal.pone.0156060&type=printable |
| 49. | Patel SK, Saggurti N, Pachauri S, Prabhakar P. Correlates of mental depression among female sex workers in Southern India. Asia Pac J Public Health. 2015;27(8):809–19. https://www.ncbi.nlm.nih.gov/pubmed/26307144 |
| 50. | Patra RK, Mahapatra B, Kovvali D, Proddutoor L, Saggurti N. Anal sex and associated HIV-related sexual risk factors among female sex workers in Andhra Pradesh, India. Sex Health. 2012;9(5):430–7. http://www.publish.csiro.au/sh/pdf/SH11155 |
| 51. | Prabhakar P, Patel SK, Saggurti N. Financial inclusion of marginalized key populations in southern India. Indian J Econ Devel. 2014;2(4):98–102. http://ijed.informaticspublishing.com/index.php/ijed/article/view/58150/45429 |
| 52. | Punyam S, Pullikalu RS, Mishra RM, Sandri P, Mutupuru BP, Kokku SB, Parimi P. Community advocacy groups as a means to address the social environment of female sex workers: a case study in Andhra Pradesh, India. J Epidemiol Community Health. 2012;66 Suppl 2:ii87–94. http://jech.bmj.com/content/jech/66/Suppl_2/ii87.full.pdf |
| 53. | Lakshmi Bai R. Crisis interventional efforts among sex workers in Tamilnadu. Int J law Psychol Human Life. 2012;1(1):1–6. |
| 54. | Rai S, Mahapatra B, Sircar S, Raj PY, Venkatesh S, Shaukat M, Rewari BB. Adherence to antiretroviral therapy and its effect on survival of HIV-infected individuals in Jharkhand, India. PLoS One. 2013;8(6):e66860. http://journals.plos.org/plosone/article/file?id=10.1371/journal.pone.0066860&type=printable |
| 55. | Raj Y, Sahu D, Pandey A, Venkatesh S, Reddy D, Bakkali T, Das C, Singh KJ, Kant S, Bhattacharya M, et al. Modelling and estimation of HIV prevalence and number of people living with HIV in India, 2010–2011. Int J STD AIDS. 2016;27(14):1257–66. https://www.ncbi.nlm.nih.gov/pubmed/26494704 |
| 56. | Ramanaik S, Thompson LH, du Plessis E, Pelto P, Annigeri V, Doddamane M, Bhattacharjee P, Shaw SY, Deering K, Khan S, et al. Intimate relationships of Devadasi sex workers in South India: An exploration of risks of HIV/STI transmission. Glob Public Health. 2014;9(10):1198–210. http://www.tandfonline.com/doi/abs/10.1080/17441692.2014.948480?journalCode=rgph20 |
| 57. | Ramanathan S, Chakrapani V, Ramakrishnan L, Goswami P, Yadav D, Subramanian T, George B, Paranjape R; Consistent condom use with regular, paying, and casual male partners and associated factors among men who have sex with men in Tamil Nadu, India: findings from an assessment of a large-scale HIV prevention program. BMC Public Health. 2013;13:827. https://www.ncbi.nlm.nih.gov/pmc/articles/PMC3854867/pdf/1471-2458-13-827.pdf |
| 58. | Ramanathan S, Deshpande S, Gautam A, Pardeshi DB, Ramakrishnan L, Goswami P, Adhikary R, George B, Paranjape RS, Mainkar MM. Increase in condom use and decline in prevalence of sexually transmitted infections among high-risk men who have sex with men and transgender persons in Maharashtra, India: Avahan, the India AIDS Initiative. BMC Public Health. 2014;14:784. https://bmcpublichealth.biomedcentral.com/track/pdf/10.1186/1471-2458-14-784?site=bmcpublichealth.biomedcentral.com |
| 59. | Ramanathan S, Nagarajan K, Ramakrishnan L, Mainkar MK, Goswami P, Yadav D, Sen S, George B, Rachakulla H, Subramanian T, et al. Inconsistent condom use by male clients during anal intercourse with occasional and regular female sex workers (FSWs): survey findings from southern states of India. BMJ Open. 2014;4(11):e005166. https://www.ncbi.nlm.nih.gov/pmc/articles/PMC4244455/pdf/bmjopen-2014-005166.pdf |
| 60. | Ramesh S, Ganju D, Mahapatra B, Mishra RM, Saggurti N. Relationship between mobility, violence and HIV/STI among female sex workers in Andhra Pradesh, India. BMC Public Health. 2012;12:764. https://bmcpublichealth.biomedcentral.com/track/pdf/10.1186/1471-2458-12-764?site=bmcpublichealth.biomedcentral.com |
| 61. | Ramesh S, Mehrotra P, Mahapatra B, Ganju D, Nagarajan K, Saggurti N. The effect of mobility on sexual risk behaviour and HIV infection: a cross-sectional study of men who have sex with men in southern India. Sex Transm Infect. 2014;90(6):491–7. http://sti.bmj.com/content/sextrans/90/6/491.full.pdf |
| 62. | Ramesh S, Mehrotra P, Saggurti N. Contributions of an intensive HIV prevention programme in increasing HIV testing among men who have sex with men in Andhra Pradesh, India. Global Public Health. 2015;10(4):474–84. http://www.tandfonline.com/doi/abs/10.1080/17441692.2014.1003571?journalCode=rgph20 |
| 63. | Ranebennur V, Gaikwad S, Ramesh S, Bhende A. Addressing vulnerabilities of female sex workers in an HIV prevention intervention in Mumbai and Thane: experiences from the Aastha project. HIV AIDS. 2014;6:9–18. https://www.ncbi.nlm.nih.gov/pmc/articles/PMC3933664/pdf/hiv-6-009.pdf |
| 64. | Rao VT, Mahapatra B, Juneja S, Singh IR. Evaluating the McDonald’s business model for HIV prevention among truckers to improve program coverage and service utilization in India, 2004–2010. HIV AIDS. 2013;5:51–60. https://www.ncbi.nlm.nih.gov/pmc/articles/PMC3576884/pdf/hiv-5-051.pdf |
| 65. | Roy KP, Mahapatra B, Bhanot A, Kapoor A, Ward D, Narayanan SS. Psychosocial correlates of HIV-related sexual risk factors among male clients in Southern India. Int J Psychol Behav Sci. 2012;2. http://www.psi.org/wp-content/uploads/drupal/sites/default/files/publication_files/International%20Journal%20of%20Psychology%20and%20Behavioral%20Sciences%202,012%20-%20Roy%20et%20al%20-%20Psychosocial%20Correlates%20of%20HIV-related%20Sexual%20Risk%20Factors%20among%20Male%20Clients%20in%20Southern%20India.pdf |
| 66. | Sadhu S, Manukonda AR, Yeruva AR, Patel SK, Saggurti N. Role of a community-to-community learning strategy in the institutionalization of community mobilization among female sex workers in India. PLoS One. 2014;9(3):e90592. http://journals.plos.org/plosone/article/file?id = 10.1371/journal.pone.0090592&type = printable |
| 67. | Saggurti N, Sabarwal S, Verma RK, Halli SS, Jain AK. Harsh realities: Reasons for women’s entry into sex work in India. J AIDS HIV Res. 2011;3:172–9. file:///C:/Users/DF/Downloads/Harsh_realities_Reasons_for_womens_involvement_in.pdf |
| 68. | Saggurti N, Jain AK, Sebastian MP, Singh R, Modugu HR, Halli SS, Verma RK. Indicators of mobility, socio-economic vulnerabilities and HIV risk behaviours among mobile female sex workers in India. AIDS Behav. 2012;16(4):952–9. https://www.ncbi.nlm.nih.gov/pmc/articles/PMC3618410/pdf/10461_2011_Article_9937.pdf |
| 69. | Saggurti N, Mahapatra B, Swain SN, Jain AK. Male migration and risky sexual behavior in rural India: is the place of origin critical for HIV prevention programs? BMC Public Health. 2011;11 Suppl 6:S6. https://www.ncbi.nlm.nih.gov/pmc/articles/PMC3287559/pdf/1471–2458-11-S6-S6.pdf |
| 70. | Saggurti N, Mishra RM, Proddutoor L, Tucker S, Kovvali D, Parimi P, Wheeler T. Community collectivization and its association with consistent condom use and STI treatment-seeking behaviors among female sex workers and high-risk men who have sex with men/transgenders in Andhra Pradesh, India. AIDS Care. 2013;25Suppl 1:S55–66. https://www.ncbi.nlm.nih.gov/pmc/articles/PMC4003583/pdf/caic25_S55.pdf |
| 71. | Saggurti N, Nair S, Malviya A, Decker MR, Silverman JG, Raj A. Male migration/mobility and HIV among married couples: cross-sectional analysis of nationally representative data from India. AIDS Behav. 2012;16(6):1649–58. https://link.springer.com/article/10.1007%2Fs10461–011-0022-z |
| 72. | Saggurti N, Verma RK, Halli SS, Swain SN, Singh R, Modugu HR, Ramarao S, Mahapatra B, Jain AK. Motivations for entry into sex work and HIV risk among mobile female sex workers in India. J Biosoc Sci. 2011;43(5):535–54. https://www.cambridge.org/core/journals/journal-of-biosocial-science/article/motivations-for-entry-into-sex-work-and-hiv-risk-among-mobile-female-sex-workers-in-india/51105A1F966562B5AD54D34077BC9897 |
| 73. | Saggurti N, Verma RK, Jain A, RamaRao S, Kumar KA, Subbiah A, Modugu HR, Halli S, Bharat S. HIV risk behaviours among contracted and non-contracted male migrant workers in India: potential role of labour contractors and contractual systems in HIV prevention. AIDS. 2008;22 Suppl 5:S127–36. https://www.ncbi.nlm.nih.gov/pubmed/19098473 |
| 74. | Sahu D, Pandey A, Mishra RM, Saggurti N, Setu S, Singh IR. An appraisal of sexual behaviors, STI/HIV prevalence, and HIV prevention programs among truckers in India: a critical literature review. World J AIDS. 2014;4:206–18. https://file.scirp.org/pdf/WJA_2014061713555433.pdf |
| 75. | Sahu D, Ramesh S, Mishra RM, Srikanth Reddy K, Bharadwaj R, Saggurti N, Pandey A, Mainkar M, George B. Are truckers being over stigmatized as HIV carriers in India? Evidences from behavioral and biological cross-sectional surveys among clients of female sex workers. Open J Prev Med. 2015;5:85–91. https://file.scirp.org/pdf/OJPM_2015031114143566.pdf |
| 76. | Sahu D, Saggurti N, Mishra RM, Ganju D, Chavan LB, Pandey A. Contribution of a large-scale HIV prevention program on condom use by long-distance truck drivers in India: a decomposition analysis. National J Commun Med. 2016;7(7):627–33. http://www.njcmindia.org/home/abstrct/899/July |
| 77. | Sakthivel SP, Raj PY, Mishra S, Sharma AK. Levels, trends and inter-regional variations in transfusion transmissible infection positivity among blood donors in India: evidence from India’s National HIV Program. World J AIDS. 2015;5:217–25. https://file.scirp.org/pdf/WJA_2015092513414576.pdf |
| 78. | Salve H, Rai S, Kant S, Raj Y, Reddy D. Demographic and sexual behavior characteristics of men who have sex with men (MSM) registered in a targeted intervention (TI) program in India. World J AIDS. 2015;5:256–64. http://file.scirp.org/pdf/WJA_2015092514384843.pdf |
| 79. | Sharma V, Saggurti N, Bharat S. Association between general media exposure and sexual behavior among mobile female sex workers in India. Int J Communic Health. 2015;6:60–8. http://communicationandhealth.ro/wp-content/uploads/2015/05/VARUN-SHARMA.pdf |
| 80. | Sharma V, Saggurti N, Bharat S. Association between system reach and exposure to interventions and characteristics of mobile female sex workers in four high HIV prevalence states in India. Glob J Health Sci. 2015;7(4):83–95. https://www.ncbi.nlm.nih.gov/pmc/articles/PMC4802063/pdf/GJHS-7-83.pdf |
| 81. | Sharma V, Saggurti N, Bharat S. Health care coverage among long-distance truckers in India: an evaluation based on the Tanahashi model. HIV AIDS. 2015;7:83–94. file:///C:/Users/DF/Downloads/HIV-76416-identifying-issues-in-different-stages-of-health-care-covera_032315.pdf |
| 82. | Singh AK, Haldar P, Rai SK, Kant S. A systematic review to explore the factors related to parent to child transmission of HIV, survival and treatment provision of children with HIV in India. World J AIDS. 2015;5:245–55. https://file.scirp.org/pdf/WJA_2015092514241096.pdf |
| 83. | Somanath RP, Mishra RM, Saggurti N, Parimi P. The association between noncommercial partnerships and risk of HIV among female sex workers: evidences from a cross-sectional behavioral and biological survey in southern India. AIDS Res Treat. 2013;2013:108630. file:///C:/Users/DF/Downloads/108630.pdf |
| 84. | Suohu K, Humtsoe C, Saggurti N, Sabarwal S, Mahapatra B, Kermode M. Understanding the association between injecting and sexual risk behaviors of injecting drug users in Manipur and Nagaland, India. Harm Reduct J. 2012;9:40. https://harmreductionjournal.biomedcentral.com/track/pdf/10.1186/1477-7517-9-40?site = harmreductionjournal.biomedcentral.com |
| 85. | Suryawanshi D, Mahapatra B, Sharma V, Kumar K, Saggurti N, Bharat S. Degree of male mobility as a risk factor for HIV in high in-migration districts of Maharashtra, India. World J AIDS. 2014;4:346–55. https://file.scirp.org/pdf/WJA_2014092215501247.pdf |
| 86. | Suryawanshi D, Patel SK, Sharma V, Adhikary R, Bharat S. Association between community collectivization and mental depression among men who have sex with men in Andhra Pradesh, India. Health Care Current Rev. 2016;4:176. https://www.omicsonline.org/open-access/association-between-community-collectivization-and-mental-depressionamong-men-who-have-sex-with-men-in-andhra-pradesh-india-2375-4273-1,000,176.pdf |
| 87. | Suryawanshi D, Patel SK, Adhikary R, Bharat S. Does mass-media public communication campaign normalize discussion, attitude and behavior about condom use among married men in India? J AIDS Clin Res. 2016;7:599. https://www.omicsonline.org/open-access/does-massmedia-public-communication-campaign-normalize-discussion-attitude-and-behavior-about-condom-use-among-married-men-in-indi-2155-6113-1,000,599.pdf |
| 88. | Suryawanshi D, Sharma V, Saggurti N, Bharat S. Factors associated with the likelihood of further movement among mobile female sex workers in India: a multinomial logit approach. J Biosoc Sci. 2016;48(4):539–56. https://www.ncbi.nlm.nih.gov/pmc/articles/PMC4890344/pdf/S0021932015000267a.pdf |
| 89. | Swain SN, Saggurti N, Battala M, Verma RK, Jain AK. Experience of violence and adverse reproductive health outcomes, HIV risks among mobile female sex workers in India. BMC Publ Health. 2011;11:357. https://bmcpublichealth.biomedcentral.com/track/pdf/10.1186/1471-2458-11-357?site = bmcpublichealth.biomedcentral.com |
| 90. | Thomas M, Sahu D, Raj Y, Pandey A. A probability model for estimating the force of transmission of HIV infection and its application. Am J Math Stat. 2014;4(3):171–7. file:///C:/Users/DF/Downloads/10.5923.j.ajms.20140403.06%20(2).pdf |
| 91. | Travasso SM, Mahapatra B, Saggurti N, Krishnan S. Non-paying partnerships and its association with HIV risk behavior, program exposure and service utilization among female sex workers in India. BMC Public Health. 2014;14:248. https://www.ncbi.nlm.nih.gov/pmc/articles/PMC3995596/pdf/1471–2458–14-248.pdf |
| 92. | Vejella S, Patel SK, Saggurti N, Prabhakar P. Community collectivization and consistent condom use among female sex workers in southern India: evidence from two rounds of behavioral tracking surveys. AIDS Behav. 2016;20(4):776–87. https://www.ncbi.nlm.nih.gov/pmc/articles/PMC4799261/pdf/10461_2015_Article_1171.pdf |
| 93. | Verma R, Shekhar A, Khobragade S, Adhikary R, George B, Ramesh BM, Ranebennur V, Mondal S, Patra RK, Srinivasan S, et al. Scale-up and coverage of Avahan: a large-scale HIV-prevention programme among female sex workers and men who have sex with men in four Indian states. Sex Transm Infect. 2010;86 Suppl 1:i76–82. http://sti.bmj.com/content/sextrans/86/Suppl_1/i76.full.pdf |
| 94. | Verma RK, Saggurti N, Singh AK, Swain SN. Alcohol and sexual risk behavior among migrant female sex workers and male workers in districts with high in-migration from four high HIV prevalence states in India. AIDS Behav. 2010;14 Suppl 1:S31–39. https://www.ncbi.nlm.nih.gov/pmc/articles/PMC3664742/pdf/10461_2010_Article_9731.pdf |
| 95. | Yadav D, Ramanathan S, Goswami P, Ramakrishnan L, Saggurti N, Sen S, George B, Paranjape R. Role of community group exposure in reducing sexually transmitted infection-related risk among female sex workers in India. PLoS One. 2013;8(10):e78361. http://journals.plos.org/plosone/article/file?id=10.1371/journal.pone.0078361&type=printable |
